# Supplementary figures and images for: The evolution of behavioral cues and signaling in displaced communication
Source: PLoS Comput Biol. 2023 Mar 27;19(3):e1010487. doi: 10.1371/journal.pcbi.1010487 (PMC10079217; doi:10.1371/journal.pcbi.1010487)

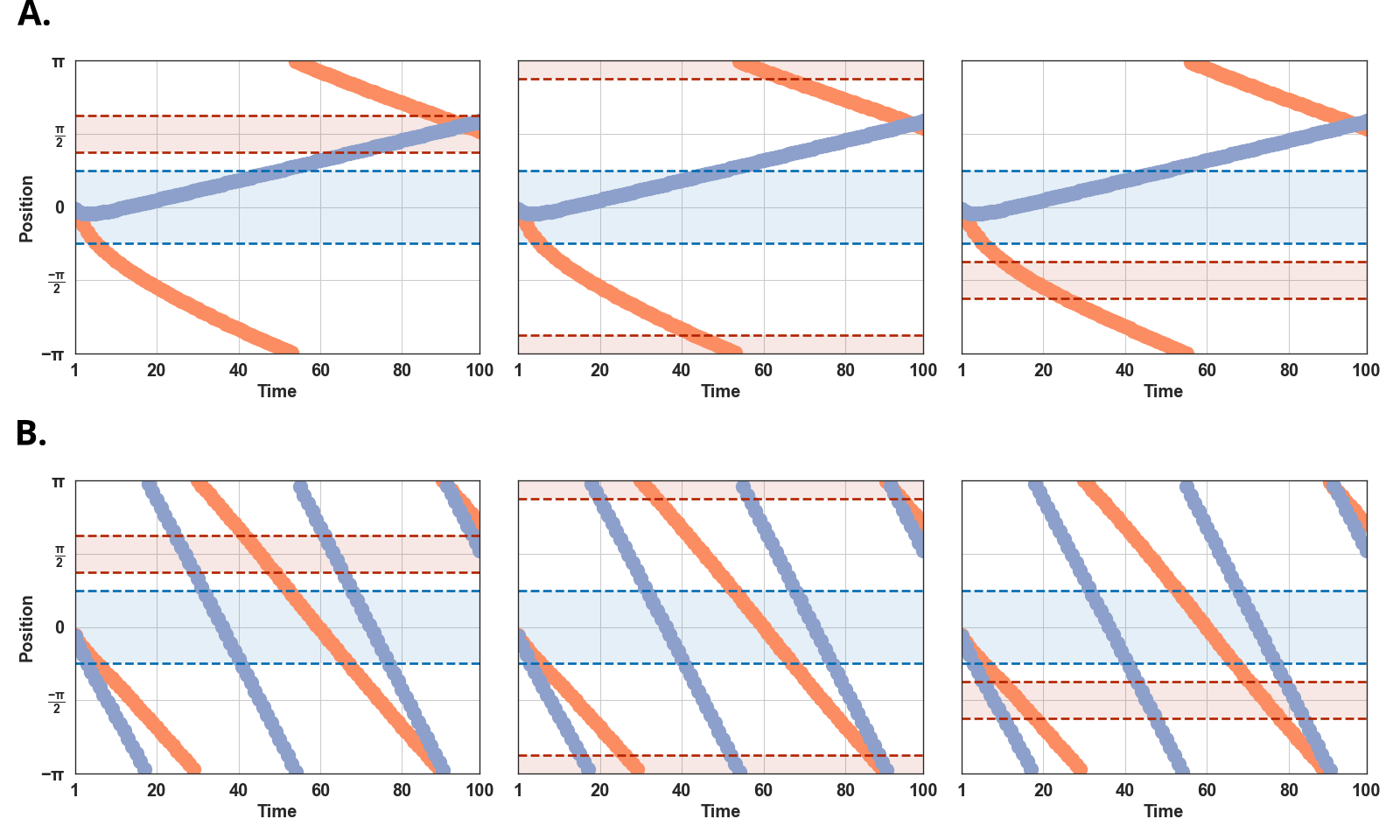

Supplement: S1 Fig — Behaviors of best performing pairs of sender and receiver in the no communication treatment for 3 given trials (i.e. 3 different food locations). Each column corresponds to a different trial. The figures show the position of the sender (resp. receiver) in red (resp. blue) at each of the 100 time steps of the trials. The position on the circle is indicated as the angular position in range [-π, π]. The communication area is indicated in blue and the foraging site containing food in red. The behaviors of two different pairs of sender and receiver are shown here. In (A), the receiver goes to the same foraging site at each trial (i.e. the foraging site located at π/2) while in (B), the receiver moves through every foraging site during the last 20 steps of simulation. (TIF) [file pcbi.1010487.s001.tif]

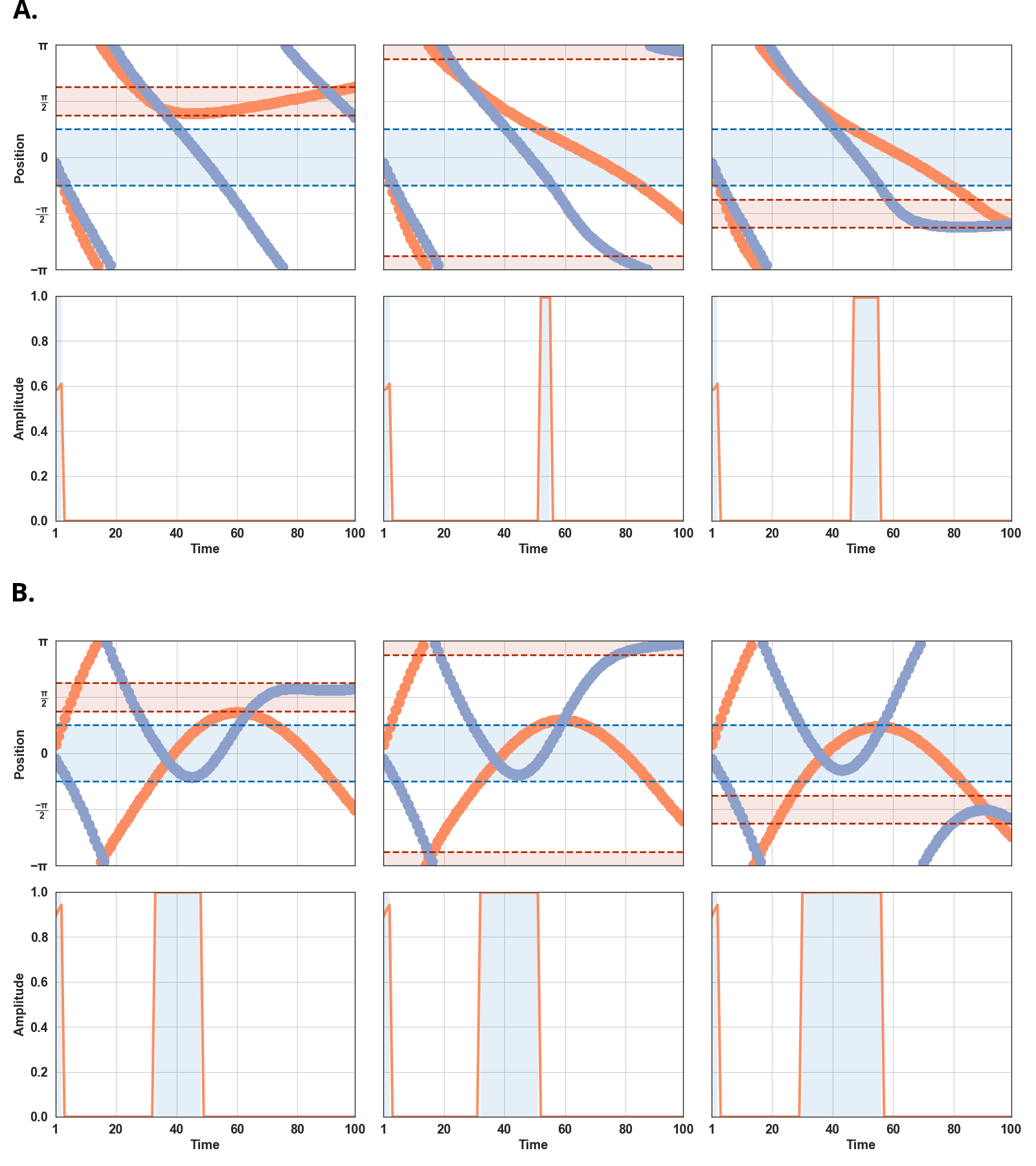

Supplement: S2 Fig — Behaviors of best performing pairs of sender and receiver in the unconstrained treatment for 3 given trials (i.e. 3 different food locations). Each column corresponds to a different trial. The top 3 figures show the position of the sender (resp. receiver) in red (resp. blue) at each of the 100 time steps of the trials. The position on the circle is indicated as the angular position in range [-π, π]. Communication area is indicated in blue and the foraging site containing food in red. The bottom 3 figures display the signal amplitude perceived by the receiver while in the communication area (blue area). The behaviors of two different pairs of sender and receiver are shown here. In (A), the pair uses onset-delay as the way to commmunicate while in (B) they use length of signaling to transmit information. (TIF) [file pcbi.1010487.s002.tif]

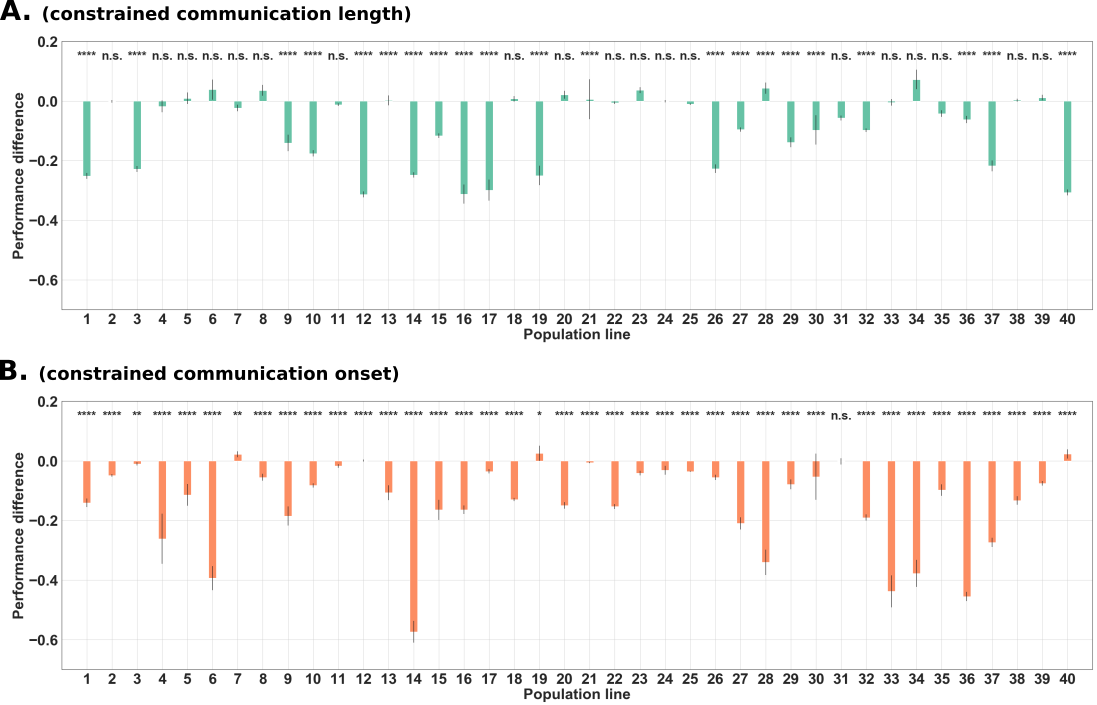

Supplement: S3 Fig — Performance difference of every population line when (A) communication length was constrained and (B) communication onset was constrained. (TIF) [file pcbi.1010487.s003.tif]

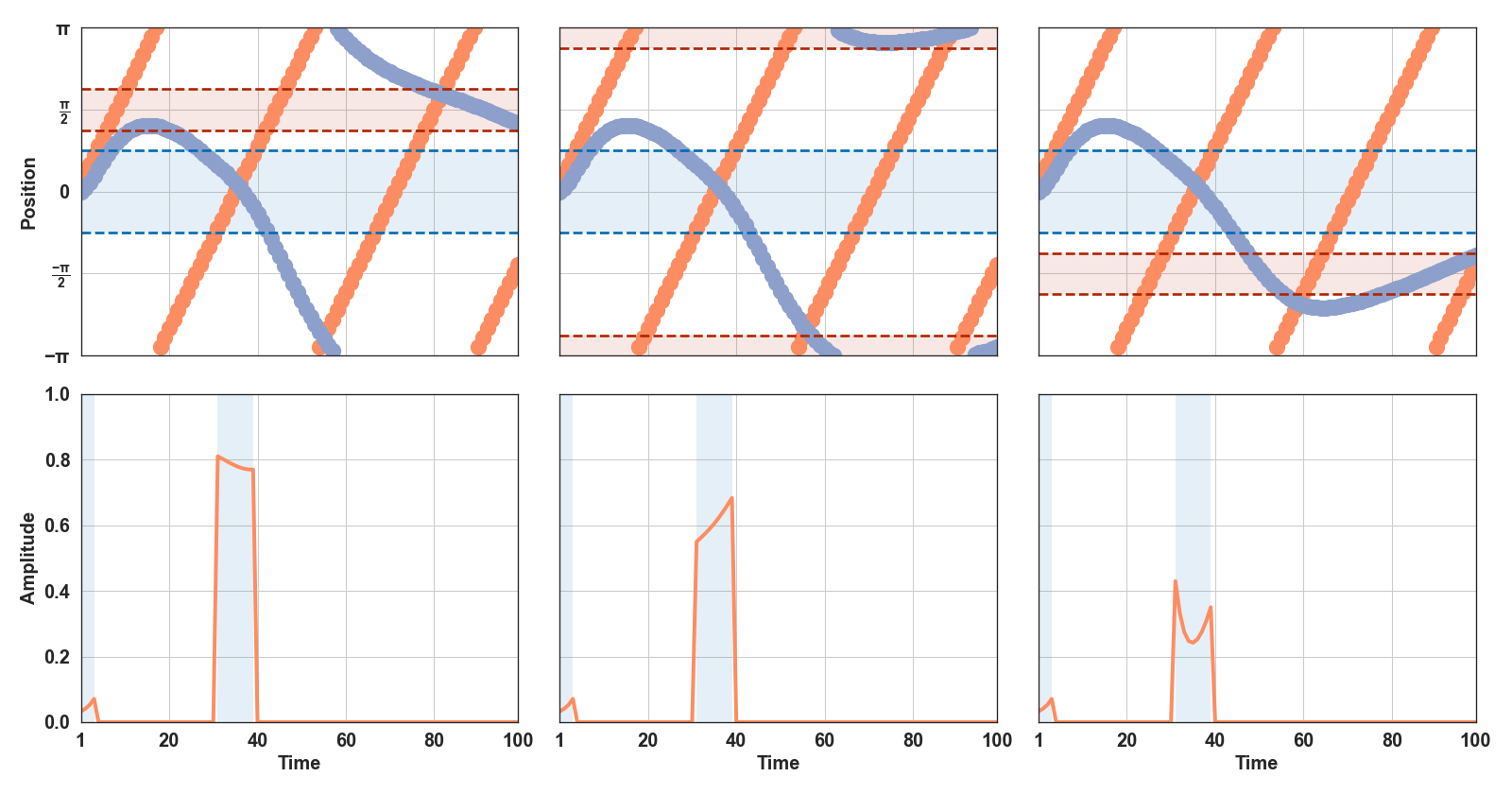

Supplement: S4 Fig — Behavior of a best performing pairs of sender and receiver in the treatment where sender velocity was constrained for 3 given trials (i.e. 3 different food locations). Each column corresponds to a different trial. The top 3 figures show the position of the sender (resp. receiver) in red (resp. blue) at each of the 100 time steps of the trials. The position on the circle is indicated as the angular position in range [-π, π]. Communication area is indicated in blue and the foraging site containing food in red. The bottom 3 figures display the signal amplitude perceived by the receiver while in the communication area (blue area). (TIF) [file pcbi.1010487.s004.tif]
