# Supplementary material for: The evolution of behavioral cues and signaling in displaced communication
Source: PLoS Comput Biol. 2023 Mar 27;19(3):e1010487. doi: 10.1371/journal.pcbi.1010487 (PMC10079217; doi:10.1371/journal.pcbi.1010487)
Supplement: Attachment — Submitted filename: Response_to_reviewers.pdf [file pcbi.1010487.s010.pdf]

Response to Comments by Reviewers

Dear Editors,

We thank you, and the reviewers, for your insightful comments and critics on our Manuscript PCOMPBIOL-D-22-01238. As per your comments, we made substantial changes to the manuscript, expanding on the description of our methodology, modifying our claims and adding more figures and supporting information to help readers better understand our work. We believe that these modifications significantly improved the quality of the manuscript and we hope you find this new version suitable for publication in PLOS Computational Biology. We thank you very much for your attention in this matter.

We follow with a detailed response to each of the comments (original comments from the reviewers are in bold for clarity). All modifications in the new version of the manuscript are tracked.

Sincerely,

Arthur Bernard, Steffen Wischmann, Dario Floreano, Laurent Keller

REVIEWER 1

it is said displaced communication is "one of the key innovations of human language", yet also stated it occurs in a few animal species. Perhaps it should be called a "feature" instead of an innovation (or qualified). On the other hand, it's "just" the abstract, so perhaps this is ok.

We agree that the wording may have been misleading and followed the reviewer's advice on replacing "innovation" by feature (line 15).

"line 52 - "defining feature of human language" -> I find that statements of this sort are either redudant or, undesirably, conceptually problematic. Is human language without displaced communication not human language anymore? Is it a claim that human language - whatever the authors' definition - would not have evolved without that feature?

We agree with the reviewer that the statement is problematic. We now have rewritten this statement as follows (lines 52 – 54):

"Displaced communication is very common in humans [5] and relatively rare in other organisms. It has also been documented in a few species such as chimpanzees [6, 7], dolphins [8] and parrots [9]."

I I fully agree with the authors' conclusions that latching onto an pre-existing behavior explains why populations do not use amplitude as cues (or, to generalize, why they do not use new behaviors as cues when completely unconstrained). But this seems to yield a claim that whatever is already there is co-opted if informative enough (vs. some other more (or equally) informative but unprecedented behavior.) While this makes sense all else being equal, I'm not entirely sure this is the case. Is there a (theoretical or practical) performance treshold above which popoulations would adopt "amplitude"? Would perhaps a performance level that makes up for the "valley of low performance values" make populations more likely to switch? In other words, what gets highly efficient, not obvious communication strategies off the ground?

This is an interesting issue. In principle, the performance level that a given system of communication may reach should not, per se, determine how likely it is to evolve. This is because evolution selects variants that provide an immediate benefit. It would be necessary to have a mechanism that foresees and selects mechanisms with "future benefits" to be able to pass through a valley of "low performance value". There has been quite a bit of discussion on this topic, starting with Sewall Wright's "shifting balance theory" of evolution. Wright pointed out that species find and ascend global peaks because evolution involves two additional processes: genetic drift (random change in allele frequency in finite populations) and inter-demic selection (wherein some local populations perform better, and so presumably produce more migrants, than do others). There is currently some discussion on the shifting balance theory, but this goes beyond the scope of the present paper as this requires either analytical analyses or simulations. We give some relevant references below:

- Wright, S. Evolution in Mendelian populations. Genetics 16, 97–159 (1931).
- Wright, S. The roles of mutation, inbreeding, crossbreeding, and selection in evolution. Proc. 6th Int. Cong. Genet. 1, 356–366 (1932)
- Maynard Smith, J. Natural selection and the concept of a protein space. Nature 225, 563–564 (1970)

- H Allen Orr. Fitness and its role in evolutionary genetics. Nat Rev Genet. 2009. 10, 531–539.
- Holzman R. et al. 2022. A new theoretical performance landscape for suction feeding reveals adaptive kinematics in a natural population of reef damselfish. Journal of Experimental Biology: doi:10.1242/jeb.243273

Another challenge is generalizing this to displaced communication as a feature of human language, in which (in the overwhelming majority of cases) there is no deterministic relationship between what is being communicated and concurrent physical cues. Perhaps the claims here should be more circumscribed to cases such as the one covered by the experiments: "remote/obscured object of interest", and not as general as "subject of communication is remote is space and/or time", which seems fairly trickier.

We agree with the reviewer that we should be more specific in our claims. We have now given a more specific definition of displaced communication as a situation *“where individuals communicate on remote or non-visible objects or organisms”* (lines 50 – 51).

overall, I find this paper very good and clever, and definitely worthy of publication in this journal. its title and some of the claims might be too ambitious, as pointed out earlier, but the work contained in the paper is at the forefront of how evolutionary robotics & agent-based modelling can tell us a lot about communication (and indeed ultimately language, though the picture is of course extremely complex).

The methods seem adequate and more advanced than those found in papers with a similar scope. it would be interesting to see a leaning into population dynamics in the future.

We thank the reviewer for their positive review of our work.

A minor revision qualifying the claims made about human language and displaced communication at large with make the paper stronger and less open to criticism which would obscure these strengths

As explained above, we have followed the reviewer’s suggestions.

suggestion on figures: I would add a bit more information to the legends, namely what is being constrained (for example, onset delay/duraton in Fig 6). It makes sense when reading the paper as it is, but I believe that being more informative will help readers looking for a quick reference

We thank you for these remarks. We added some clarification in the legends of multiple Figures in the revised version of the manuscript. Legend of Figure 2 was altered as follows (lines 113 – 114):

“by setting the signal perceived by the receiver to a constant zero value”

We also modified the legend of Figure 3 (lines 138 – 139):

“In the no-communication treatment, receivers perceived a signal with a constant zero value.”

Finally, the legend of Figure 7 (following the numbering of the revised version, previously Figure 6) was altered (lines 274 – 275):

“In the constrained treatment, senders were forced to move at a fixed velocity, ...”

I would also recommend being precise about experiments/figures when referring to them (e.g. lines 295-296: "in the experiments where agents were constrained to use signal amplitude there there was [...]").

Following the reviewer's suggestion, we altered the text to be more precise. We added the following section in the Discussion (lines 311 – 314 and lines 320 - 325):

“Our time-course analyses confirmed this hypothesis, showing that on average it took 42 times more generations for communication to evolve (i.e., for populations to reach a higher performance than that attained by populations where communication of any form was impossible) via signal amplitude than via signal onset-delay/duration.”

“In line with this view of a slow stochastic process being required for reaching a difficult evolutionary target, we showed that there was a large variation across populations in the number of generations required to surpass a performance score of 0.2 (the performance hallmark of some form of communication) when agents could not use signal onset-delay/duration to communicate and had to rely on signal amplitude.”

REVIEWER 2

General remarks:

- At the end of the introduction, it is not clear to me what the receiver agent does with the information received from the sender (the information seems to be missing), which is problematic before going to the results section.

Thank you for pointing this out. It is true the description of the experimental setting was not clear. We reworded this section as follows (lines 70 – 79):

"The performance of each pair of sender-receiver agents was evaluated as the proportion of the time spent by the receiver on the foraging site containing food. Importantly, the sender, but not the receiver, could perceive the presence of food when at a site containing food. Communication between the sender and receiver was possible only when they were simultaneously in the communication area in the nest. There, the sender had the possibility to produce a signal whose amplitude could vary continuously and the receiver could potentially use this information to infer which of the foraging sites contained food. The performance of each pair of sender-receiver agents was evaluated in the last 20 time steps (out of 100) of each trial as the proportion of the time spent by the receiver on the foraging site containing food."

- How are the main claims (e.g. signal amplitude vs others) influenced by the neural network type used? This is an important point which should be clearly discussed and compared to what was done in other studies. Why CTRNNs? and not usual RNN, GRU or LSTM?

As the reviewer correctly pointed out, there are numerous types of neural network architectures and activation functions that can capture --and respond to-- time-dependent information. For the purpose of the questions addressed in this study, we wanted to exclude the possibility that agents could not, at least in principle, capture and respond to time-dependent information. In this perspective, any other type of neural network that can capture and respond to time-dependent information could be used. These and other neural network parameters (e.g., number of hidden units and number of layers, range of connection weights, input and output encoding) may affect the genome size, search space, and maximum performance values, but would equally affect all treatment conditions. The neural network used in these experiments is a recurrent neural network (RNN) where the activation function is continuously updated in time (CTRNN), thus making it more general than discrete-time recurrent neural networks, as discussed in reference [37]. This type of neural network has often been used in evolutionary experiments resorting to agents with time-dependent behavior and in a previous study on the evolution of displaced communication [24]. We have better explained these points in the Materials and Methods section (lines 383 – 391):

"Each agent was controlled by an individual neural network that, given a set of inputs representing the sensory information of the agent, computed its desired behavior at each time step. In particular, agents were endowed with continuous-time recurrent neural networks (CTRNN) [37] (Fig 8), which have been frequently applied in evolutionary robotics [24, 38]. In contrast to feedforward neural networks, CTRNN form a directed acyclic graph which allows them to store an internal state akin to a memory. This enables the network to display dynamic temporal behavior and act on inputs removed in time, a feature especially suited for the evolution of displaced communication. To compute the activation of its neurons, a CTRNN uses an ordinary differential."

- How the hyperparameter were chosen? (mutation rate, number of agents, population size, ...). Are they standard values used in other studies?

Yes, the hyperparameters chosen in this study are similar to those used in other evolutionary experiments conducted in simulation. Different hyperparameter values would affect the increment rate of foraging performance in all treatments but there would be no impact on the comparative outcomes and conclusions of the article.

- Figures could be more informative and more Figures should be provided, in particular explanatory schemas: different types of communication, example of the steps of how agents behave (first sender look at where is the food before locating the receiver, etc.), which operations done on genes, etc.).

We agree and added several figures and supporting information to help readers better understand our findings. First, we added a new explanatory Figure (Fig 4) illustrating the different modes of communication (signal amplitude, onset-delay and signal duration) presented in the manuscript. In addition, we added multiple supplementary Figures and Videos to illustrate the behaviors of evolved pairs of individuals as Supporting Information. S1 Fig and S1 & S2 Videos illustrate the two different behaviors evolved during the no-communication treatment presented in the manuscript. S2 Fig and S3 & S4 Videos display the evolved behaviors in the unconstrained treatment, illustrating the use of onset-delay and signal duration to communicate. Finally, S4 Fig and S5 Video show the behavior of a pair of sender and receiver using signal amplitude as a way to communicate in the unconstrained treatment.

- In general, there is a lack summarization of experimental design and several methodological details are missing (CTRNN is mentioned once and no details are provided). This does not able to reader to properly evaluate if the results are interesting. More general information should be provided, including for people outside the field of communication evolution with NN-based agents.

We thank the reviewer for highlighting the lack of details in our Methods section and potential problems for readers outside of the field. We have now rewritten the section on the description of the neural network design to give a more thorough description of the setting: (lines 383 – 410):

"Each agent was controlled by an individual neural network that, given a set of inputs representing the sensory information of the agent, computed its desired behavior at each time step. In particular, agents were endowed with continuous-time recurrent neural networks (CTRNN) [37] (Fig 8), which have been frequently applied in evolutionary robotics [24, 38]. In contrast to feedforward neural networks, CTRNN form a directed acyclic graph which allows them to store an internal state akin to a memory. This enables the network to display dynamic temporal behavior and act on inputs removed in time, a feature especially suited for the evolution of displaced communication. To compute the activation of its neurons, a CTRNN uses an ordinary differential. Activation of a given neuron i at time step t is computed following the Euler step as follows:

$y_{it} = \sigma(s_{it} + \theta_i)$,

where σ is the sigmoid activation function, θ_i the bias term for neuron i *and s_{it} the state of neuron* i *at time step* t. *s_{it} is calculated as:*

$s_{it} = \alpha * \tau_i * (\sum_j W_{ji} * y_{jt} - s_{i(t-1)})$,

where α is the step size (0.1 in our experiments), τ_i is the time constant for neuron i, $\mathrm{W_{ji}}$ *the connection weight from neuron* j *to neuron* i, y_{jt} *the activation of neuron* j *at time step* t *and* $\mathrm{s_{i(t-1)}}$ *the stage of neuron* i *at time step* $t-1$.

Both senders and receivers possessed two inputs indicating their own location (expressed as sine and cosine values). The sender had an additional input providing information on whether the foraging site on which it was contained food (1 if food was present, 0 otherwise) and the receiver had an additional input corresponding to the perceived signal amplitude. Each network included five hidden neurons with recurrent connections and two output neurons. The two output neurons controlled the speed and direction of the agent. Speed was computed as the absolute value of the difference between these two outputs and direction as the sign of this difference. In addition, the sender had a supplementary output encoding the signal amplitude. Neural networks were allowed to evolve via the connection weights between neurons"

We also separated Figure 1 (illustration of experimental setting) in two so that a new separate Figure, Figure 7, can illustrate on its own the neural controllers of the agents.

Small Remarks:

- This does not correspond to figure 1: (line 78-79) "The specifications of the agents' neural networks were encoded in an artificial genome (Fig 1). "

We agree that Figure 1 did not clearly provide the information we wanted to convey. We have therefore created a new figure (Figure 7) to illustrate the neural network controllers of the agents.

- Some sentences are not undertandable: 156-157: "... provided by variation in when the signal was ...""

Thank you for pointing out this. We modified multiple unclear sentences in the manuscript including the one mentioned by the reviewer (lines 163 – 167):

"Thus, information on food location could be provided to the receiver either by the delay between the time when the signal was first perceived by the receiver in the nest and the start of the trial (i.e., onset-delay, Fig 4B) or by the amount of time when both the sender and the receiver were simultaneously in the nest (i.e., signal duration, Fig 4C)."

- The GitHub is provided, which is good, but the readme is kind of short. The authors should provide more details on how to use their code, and in particular how to reproduce their figures and provide the random seeds used.

The reviewer is right in pointing that the GitHub was not sufficiently documented to allow readers to reproduce our figures. Moreover, the repository was lacking the proper scripts used to create the figures. We added the script and completed the Readme file in accordance with these additions. As for the random seeds, they are all present with the data on the Dryad Digital Repository. Each population line of every experiment is provided with the random seed that was used.

- Evolutionary data link is provided, but it seems that the corresponding DOI does not exist: https://doi.org/10.5061/dryad.f4qrfj6zt

The DOI does not exist until the paper is published and the data made publicly available. In the meantime, you can find a reviewer accessible version at this link: https://datadryad.org/stash/share/E3eZe_LWQYm6X7PT0vbMdl2BLDWAicL1czFyzJ6scoY
