# Supplementary material for: The evolution of behavioral cues and signaling in displaced communication
Source: PLoS Comput Biol. 2023 Mar 27;19(3):e1010487. doi: 10.1371/journal.pcbi.1010487 (PMC10079217; doi:10.1371/journal.pcbi.1010487)
Supplement: Attachment — Submitted filename: Response_to_reviewers.pdf [file pcbi.1010487.s011.pdf]

# Response to Comments by Reviewers

Dear Editors,

We thank you, and the reviewers, for the insightful comments on our Manuscript PCOMPBIOL-D-22-01238. As per your comments, we made changes to the manuscript to improve the overall clarity of our work. We believe that these modifications significantly improved the quality of the manuscript and we hope you find this new version suitable for publication in PLOS Computational Biology. We thank you very much for your attention in this matter.

We follow with a detailed response to each of the comments (original comments are in bold for clarity). All modifications in the new version of the manuscript are tracked.

Sincerely,

Arthur Bernard, Steffen Wischmann, Dario Floreano, Laurent Keller

**1) In the main paper text, it is easy for the reader to miss the crucial specification (line 68) that the agents are moving in a *one dimensional* circular environment, i.e. only along the perimeter of the 'arena' illustrated in figure 1. Nowhere else in the subsequent description or caption to figure 1 (not until the detailed methods, or supplementary videos) is it made clear that the agents move by varying their angular velocity (speed and direction). Consequently it is hard to follow what behaviour is exhibited by the agents or why, for example, communication onset or duration can be constrained by fixing the sender agent's velocity. Please add a few explanatory phrases about the agent's movement to the main text introducing the environment and to the caption of figure 1.**

We agree with the editor that the manuscript was lacking sufficient details on the specifics of the environment before moving to the Results section. We modified the introduction as follows (lines 68-72):

*"In each experiment, a signal sender and a signal receiver were placed on a one-dimensional circular environment containing a region ("nest") where they could communicate, and five non-overlapping foraging sites, only one of which contained food at any given time (Fig 1). Agents could freely move clockwise and counterclockwise on the perimeter of this circle by varying their angular velocity."*

We also further reinforced this point in the caption of Figure 1 (lines 95-101):

*"At each trial, food was randomly located on one of the five foraging sites (marked in red) equally spaced on a one-dimensional circle. Both the sender and the receiver always started a trial at position 0 on the circle located within the communication area, which acted as a "nest" (marked in blue). Agents moved on the border of the circular environment in a direction or the other (clockwise or counterclockwise). See Materials and Methods for a complete description of the experimental setup."*

**2) The definition of 'onset-delay' is confusingly phrased (line 164, caption to figure 4) as "the varying delay between the time when the signal was first perceived by the receiver in the nest and the start of the trial". This implies 'first perceived' happens before 'start of trial'. Please revise, where-ever it occurs to "the varying delay from the start of the trial to the time when the signal was first perceived by the receiver in the nest".**

We thank you for pointing the confusion that stems from this phrasing. We modified the main text accordingly in two parts of the manuscript (lines 168-170 and lines 195-197):

*"Thus, information on food location could be provided to the receiver either by the delay from the start of the trial to the time when the signal was first perceived by the receiver in the nest (i.e., onset-delay, Fig 4B)"*

*"B) When using onset-delay, the varying delay from the start of the trial to the time when the signal was first perceived by the receiver in the nest provides information on food location."*

**3) Line 333, it is not clear why "changes in either the signaling or response strategy would [necessarily] destroy the communication system that is already in place". Indeed it seems very plausible that the existence of one strategy could bootstrap the development of another, more efficient strategy, with both co-existing until the more efficient one takes over. Possibly this did not happen in the current simulation because**

**the potential increase in performance (from 0.47 to 0.51) is relatively small, so much longer evolutionary time would be required to discover it. Please revise the discussion to address this point.**

That is an interesting remark. Our point was that if some individuals change their signaling or response strategy in a population where a system of communication is relatively efficient this is likely to lead to a decreased performance for these individuals. We changed the section (lines 341-349) to clarify that:

*“This may account for some of the differences in signaling observed between closely related species and isolated populations of a given species. For example, Anolis lizards originating from different evolutionary ancestors have evolved different signaling systems in response to similar selective pressures [32]. It is also possible that a new mode of communication could evolve and coexist with the original mode of communication but this would probably require that it does not interfere with it. This is because once a mode of communication has evolved, individuals changing their signaling or response strategy are likely to have lower performance. But new modes of communication may evolve when this does not lead to a disruption of the original mode of communication.”*
